# Prenatal Exposure to Severe Stress and Risks of Ischemic Heart Disease and Stroke in Offspring

**DOI:** 10.1001/jamanetworkopen.2023.49463

**Published:** 2023-12-27

**Authors:** Fen Yang, Imre Janszky, Nathalie Roos, Jing Li, Krisztina D. László

**Affiliations:** 1 Department of Global Public Health, Karolinska Institutet, Stockholm, Sweden; 2 Department of Public Health and Nursing, Norwegian University of Science and Technology, Trondheim, Norway; 3 Division of Clinical Epidemiology, Department of Medicine Solna, Karolinska University Hospital, Karolinska Institutet, Stockholm, Sweden; 4 Department of Clinical Medicine–Department of Clinical Epidemiology, Aarhus University Hospital, Aarhus, Denmark; 5 Department of Public Health and Caring Sciences, Uppsala University, Uppsala, Sweden

## Abstract

**Importance:**

Prenatal stress is associated with increased risks of several cardiovascular risk factors later in life. However, knowledge regarding the role of prenatal stress in the development of ischemic heart disease (IHD) and stroke is very limited.

**Objective:**

To examine prenatal stress, defined as maternal bereavement, and risks of IHD and stroke in the offspring.

**Design, Setting, and Participants:**

A cohort study was conducted using data from Danish and Swedish registries. Live singleton births during calendar years 1973-2016 in Denmark (followed up until December 31, 2016) and during calendar years 1973-2014 in Sweden (followed up until December 31, 2021) were included in the analysis.

**Exposure:**

Maternal loss of a close family member (partner, older children, parents, or siblings) the year before or during the pregnancy.

**Main Outcome and Measures:**

Diagnoses of IHD and stroke. Cox proportional hazards regression models were used to estimate hazard ratios (HRs) and 95% CIs for IHD and stroke in the offspring according to maternal bereavement.

**Results:**

The study included 6 758 560 births (39.4% from Denmark; 51.4% boys). During the median follow-up of 24.6 (IQR, 13.9-35.1) years, 8664 offspring (0.1%) were diagnosed with IHD and 13 094 with stroke (0.2%). Overall, maternal bereavement the year before or during pregnancy was not associated with IHD (adjusted HR [AHR], 0.98; 95% CI, 0.85-1.13) or stroke (AHR, 1.04; 95% CI, 0.94-1.16) in offspring. Similarly, no associations were observed when exposure was classified by the mother’s relationship to the deceased individual, ie, loss of older child or partner (HR, 0.85; 95% CI, 0.64-1.14 for IHD and 0.98; 95% CI, 0.77-1.25 for stroke) or loss of parent or sibling (HR, 1.03; 95% CI, 0.87-1.21 for IHD and 1.06; 95% CI, 0.94-1.19 for stroke). However, associations between loss in the third trimester and IHD (AHR, 1.50; 95% CI, 1.06-2.13), and loss due to cardiovascular disease and stroke (AHR, 1.22; 95% CI, 1.03-1.44) were identified when exposure was classified by time of loss or the relative’s cause of death.

**Conclusions and Relevance:**

The findings of this study provide little support for the hypothesis that prenatal stress is associated with risks of IHD and stroke in the first 5 decades of life. However, the association observed between stress in the third trimester and IHD warrants further investigation.

## Key Points

**Question:**

Is prenatal stress associated with increased risks of ischemic heart disease (IHD) and stroke in the offspring?

**Findings:**

In this cohort study including 6.8 million participants from Denmark and Sweden, no association between maternal stress during pregnancy, defined as loss of a close relative, and the offspring’s risk of IHD and stroke up to middle-age was observed. However, maternal bereavement in the third trimester was associated with an increased risk of IHD.

**Meaning:**

The findings of this study suggest that severe maternal stress during pregnancy is not associated with IHD and stroke risk up to middle-age in the offspring.

## Teaser

This cohort study examines the risk of ischemic heart disease and stroke in adults who were exposed to prenatal stress when their mothers experienced the death of a close family member.

## Introduction

Ischemic heart disease (IHD) and stroke, 2 major types of cardiovascular diseases (CVDs), remain the leading causes of morbidity and mortality worldwide.^1^ Despite the substantial decrease in age-adjusted mortality due to IHD and stroke in the Western world, the incidence rates of these 2 diseases have generally remained stable or even increased slightly in younger individuals.^2,3^ The etiology of IHD and stroke in younger persons is considered to be different from that in those who are older,^4,5^ and traditional cardiovascular risk factors, such as smoking, obesity, dyslipidemia, and hypertension, do not fully account for IHD and stroke in the younger population.^2,6,7^ Considering the important personal and societal socioeconomic impact of an IHD or stroke diagnosis in younger persons, including costs related to productivity loss and long-term health care, a better understanding of their etiology is needed.

According to the hypothesis of the fetal origins of adult disease, maternal exposure to stress during pregnancy and the subsequent physiologic and lifestyle changes may induce a suboptimal intrauterine environment that may lead to a dysregulation of the fetal hypothalamic-pituitary-adrenal axis.^8^ The resulting hormonal, inflammatory, and cardiometabolic environment may in turn increase the risk of adverse birth outcomes, such as fetal growth restriction^9^ and preterm birth,^10^ as well as long-term health outcomes, including neurodevelopmental^11^ and cardiometabolic^12^ disorders. Since fetal growth restriction, preterm birth, and psychiatric and cardiometabolic disorders are known risk factors for IHD and stroke,^13,14,15,16^ an association between maternal stress during pregnancy and the risk of IHD and stroke in the offspring is plausible. However, few studies have investigated these possible associations, and their findings have been inconclusive.^17,18,19,20,21^

The death of a close family member is one of the most adverse life events.^22^ The loss is particularly difficult if it concerns a member of the nuclear family, such as a child or a spouse, or if it is an unnatural, sudden death.^22^ Thus, if maternal stress during pregnancy is involved in the etiology of IHD or stroke, we may expect an association between bereavement shortly before or during pregnancy—particularly in the case of death of a nuclear family member or of a sudden loss—and the risk of IHD and stroke. A Danish cohort study reported a modestly increased risk of overall CVD among individuals prenatally exposed to maternal bereavement up to the age of 40 years,^21^ but it lacked statistical power to perform detailed analyses concerning associations for specific CVD types, such as IHD and stroke. In this cohort study, we used bereavement as an indicator of severe stress and analyzed whether maternal stress the year before or during pregnancy is associated with increased risks of IHD and stroke in the offspring up to the age of 49 years and whether risk estimates for IHD and stroke differed by the relationship to the deceased, the deceased’s cause of death, and the time of loss in relation to pregnancy.

## Methods

### Study Population and Design

We included live singleton births from the Danish Medical Birth Register (DMBR)^23^ during calendar years 1973-2016 and from the Swedish Medical Birth Register (SMBR)^24^ during calendar years 1973-2014. All residents in Denmark and Sweden are assigned a unique personal number^25^ allowing accurate linkage between registers. A detailed description of the registers from which we extracted data for this study is provided in eTable 1 in Supplement 1. After excluding offspring whose mothers had a missing or incomplete personal identification number (n = 7925) or whose mother could not be linked to any relative (n = 81 144), the study population consisted of 6 758 560 live births. We followed the Strengthening the Reporting of Observational Studies in Epidemiology (STROBE) reporting guideline for cohort studies.^26^ The study was approved by the Danish Data Protection Agency and the Research Ethics Committee at Karolinska Institute in Stockholm. The boards do not request informed consent for register-based studies.

### Measures

#### Exposure

Study participants were considered exposed if their mothers experienced the death of a close family member, that is, older child, partner (ie, the father of the index child), parent, or sibling, the year before or during pregnancy. We used the Danish Civil Registration System and the Swedish Multi-Generation Register to identify mothers’ relatives and the Danish Register of Causes of Death and the Swedish Cause of Death Register to retrieve information on deceased relatives’ date and cause of death. In case of several losses during the exposure window, we considered the first loss in the analyses. The exposure was further categorized according to (1) the time of the mother’s loss (7-12 months before pregnancy, 0-6 months before pregnancy, and first, second, and third trimesters), (2) the mother’s relationship to the deceased (older child or partner and parent or sibling), and (3) the deceased relative’s cause of death (unnatural causes [ie, suicide, homicide, accident, or other external causes], CVD, and other natural causes, including all other medical conditions), using the *International Classification of Diseases, 8th Revision* (*ICD-8*), *ICD-9*, and *International Statistical Classification of Diseases, 10th Revision* (*ICD-10*) codes reported in eTable 2 in Supplement 1).

#### Outcome

We obtained information on primary diagnoses and main causes of deaths due to IHD or stroke (including its 2 main types, ie, hemorrhagic stroke and ischemic stroke) from the Danish and Swedish patient and cause of death registers, using the *ICD-8*, *ICD-9*, and *ICD-10* codes reported in eTable 2 in Supplement 1. We followed up study participants from birth until the date of the first diagnosis of IHD or stroke, death, emigration, or the latest available date (December 31, 2016, in Denmark and December 31, 2021, in Sweden), whichever occurred first.

#### Covariates

The maternal characteristics we considered were country of origin, marital status (from the Danish Civil Registration System and the Swedish Total Population Register), highest educational level (from the Danish Integrated Database for Labour Market Research and the Swedish Register of Education), age at delivery, parity, smoking (available since 1991 in Denmark and since 1982 in Sweden) and body mass index (BMI) in early pregnancy (from the DMBR [available since 2003] and the SMBR [available since 1982]), diseases before delivery (including diabetes, hypertensive disease, and psychiatric disorders, extracted from the DNPR and the SMBR using the *ICD-8*, *ICD-9*, and *ICD-10* codes in eTable 2 in Supplement 1), and family history of CVD from the DNPR and the SPR. Race and ethnicity data were not available in Danish and Swedish registers.

Offspring characteristics we considered included sex, calendar year of birth, gestational age, and birth weight (from the DMBR and the SMBR), and diagnoses of congenital heart diseases (CHD) (from the DNPR and SPR using the *ICD-8*, *ICD-9*, and *ICD-10* codes in eTable 2 in Supplement 1). We also retrieved information on preterm birth (ie, gestational age at birth <37 weeks) and small for gestational age birth (ie, birth weight below the 10th percentile of the sex and gestational age–specific reference curve for normal fetal growth^27^).

### Statistical Analysis

We used Cox proportional hazards regression models to estimate hazard ratios (HRs) and 95% CIs for IHD and stroke (including the 2 main subtypes of stroke) according to maternal bereavement, with attained age as the underlying time scale. The proportional hazards assumption was assessed using Schoenfeld residuals; there was no evidence of violation of the proportional hazards assumption. Analyses with the follow-up time split as younger than 18 years, 18 to 30 years, and older than 30 years indicated similar results (eTable 3 in Supplement 1). We further investigated whether any association between bereavement and the risks of IHD and stroke varied by the mothers’ relationship to the deceased (older child or partner vs parent or sibling) and cause of death (unnatural death and death due to CVD or other natural death). To study the importance of the timing of bereavement, we performed analyses with exposure categorized as 7 to 12 months before pregnancy, 0 to 6 months before pregnancy, and first, second, and third trimesters of pregnancy.

We ran unadjusted and multivariate models. Our main multivariable models included offspring sex, country, and year of birth; maternal country of origin, parity, age, educational level, and marital status at delivery; and maternal hypertensive disease, diabetes, psychiatric disorders, and family history of CVD before delivery. We treated the group with missing values as a separate category. We considered confounders factors that were associated with both exposure and outcome but could not theoretically be regarded mediators. Since the timing of presentation of maternal hypertension and diabetes (potentially both confounders and mediators) in relation to exposure could not exactly be determined for all factors and as estimates in the unadjusted and multivariate models were largely similar, we chose to include maternal hypertension and diabetes before delivery in the main multivariate models. To assess whether any associations between maternal bereavement and risks of IHD and stroke differ according to offspring sex, country, or year of birth, we performed stratified analyses and formal tests of interaction with these variables.

For sensitivity analysis, we did not adjust for preterm birth, small for gestational age, or CHD in the main models since these factors may be associated with maternal bereavement and offspring CVD. Since information on maternal smoking and BMI in early pregnancy was not available for all participants, we adjusted for these measures only among those with available data.

Statistical analyses were performed using SAS, version 9.4 (SAS Institute Inc) and RStudio, version 1.2.1578 (RStudio Inc). A 2-sided *P* < .05 value was set as the threshold for significance.

## Results

Among the 6 758 560 live births (39.4% from Denmark, 60.6% from Sweden, 51.4% boys, 48.6% girls) whose mothers had register links to at least a family member, 167 192 individuals (2.5%) were exposed to maternal bereavement the year before or during pregnancy. During the median follow-up of 24.6 (IQR, 13.9-35.1) years, 8664 offspring (0.1%) were diagnosed with IHD and 13 094 were diagnosed with stroke (0.2%). Offspring with IHD or stroke were more likely to be born before 1978, to have a diagnosis of CHD, a mother younger than 19 years at delivery, and a lower educational level than study participants free of IHD and stroke (Table 1).

**Table 1.** Characteristics of the Study Population According to Ischemic Heart Disease and Stroke Diagnoses in the Offspring

Characteristic	Ischemic heart disease, No. (%)		Stroke, No. (%)	
	No	Yes	No	Yes
**Offspring characteristics**				
Study country				
Denmark	2 659 471 (99.8)	5105 (0.2)	2 659 023 (99.8)	5553 (0.2)
Sweden	4 090 425 (99.9)	3559 (0.1)	4 086 443 (99.8)	7541 (0.2)
Calendar year of birth				
1973-1978	977 882 (99.5)	5386 (0.5)	977 707 (99.4)	5561 (0.6)
1979-1984	858 344 (99.8)	1804 (0.2)	857 288 (99.7)	2860 (0.3)
1985-1990	968 138 (99.9)	837 (0.1)	966 939 (99.8)	2036 (0.2)
1991-1996	1 029 259 (99.9)	373 (0.04)	1 028 425 (99.9)	1207 (0.1)
1997-2002	887 100 (99.9)	136 (0.02)	886 672 (99.9)	564 (0.1)
2003-2008	953 290 (99.9)	80 (0.01)	952 921 (99.9)	449 (0.1)
2009-2016	1 075 883 (99.9)	48 (0.01)	1 075 514 (99.9)	417 (0.1)
Sex				
Male	3 466 707 (99.8)	5734 (0.2)	3 465 547 (99.8)	6894 (0.2)
Female	3 283 189 (99.9)	2930 (0.1)	3 279 919 (99.8)	6200 (0.2)
Congenital heart disease				
No	6 650 206 (99.9)	8153 (0.1)	6 647 027 (99.8)	11 332 (0.2)
Yes	99 690 (99.5)	511 (0.5)	98 439 (98.2)	1762 (1.8)
Preterm birth				
No	6 018 270 (99.9)	5292 (0.1)	6 013 671 (99.8)	9891 (0.2)
Yes	309 216 (99.9)	329 (0.1)	308 903 (99.8)	642 (0.2)
Unknown	422 410 (99.3)	3043 (0.7)	422 892 (99.4)	2561 (0.6)
Small for gestational age^a^				
No	5 677 729 (99.9)	4581 (0.1)	5 673 357 (99.8)	8953 (0.2)
Yes	626 707 (99.8)	1023 (0.2)	626 202 (99.8)	1528 (0.2)
Unknown	445 460 (99.3)	3060 (0.7)	445 907 (99.4)	2613 (0.6)
**Maternal characteristics**				
Country of origin same as the study country				
No	874 448 (99.9)	721 (0.1)	873 981 (99.9)	1188 (0.1)
Yes	5 863 805 (99.9)	7907 (0.1)	5 859 828 (99.8)	11 884 (0.2)
Unknown	11 643 (99.7)	36 (0.3)	11 657 (99.8)	22 (0.2)
Age at the time of the index birth, y				
≤19	202 920 (99.6)	735 (0.4)	202 801 (99.6)	854 (0.4)
20-24	1 351 463 (99.8)	2825 (0.2)	1 350 601 (99.7)	3687 (0.3)
25-29	2 395 342 (99.9)	2976 (0.1)	2 393 688 (99.8)	4630 (0.2)
30-34	1 899 326 (99.9)	1580 (0.1)	1 898 090 (99.8)	2816 (0.2)
≥35	900 845 (99.9)	548 (0.1)	900 286 (99.9)	1107 (0.1)
Level of education at the time of the index birth				
Primary and lower secondary	1 484 357 (99.7)	3876 (0.3)	1 483 646 (99.7)	4587 (0.3)
Upper secondary	3 060 526 (99.9)	3415 (0.1)	3 058 289 (99.8)	5652 (0.2)
Bachelor’s degree or higher	2 103 651 (99.9)	1232 (0.1)	2 102 215 (99.9)	2668 (0.1)
Unknown	101 362 (99.9)	141 (0.1)	101 316 (99.8)	187 (0.2)
Marital status at the time of the index birth				
Not married/not in registered partnership	3 116 362 (99.9)	2813 (0.1)	3 114 140 (99.8)	5035 (0.2)
Married/registered partnership	3 471 971 (99.9)	5176 (0.1)	3 469 964 (99.8)	7183 (0.2)
Unknown	161 563 (99.6)	675 (0.4)	161 362 (99.5)	876 (0.5)
Parity at the time of the index birth				
1	2 924 810 (99.9)	3628 (0.1)	2 922 780 (99.8)	5658 (0.2)
2	2 488 029 (99.9)	3085 (0.1)	2 486 384 (99.8)	4730 (0.2)
≥3	1 337 057 (99.9)	1951 (0.1)	1 336 302 (99.8)	2706 (0.2)
Smoking during the index pregnancy^b^				
No	3 760 262 (99.9)	759 (0.02)	3 757 945 (99.9)	3076 (0.1)
Yes	791 975 (99.9)	333 (0.04)	791 224 (99.9)	1084 (0.1)
Unknown	287 273 (99.9)	120 (0.1)	287 006 (99.9)	387 (0.1)
BMI during the index pregnancy^c^				
<18.5	126 928 (99.9)	28 (0.02)	126 806 (99.9)	150 (0.1)
18.5-24.9	1 998 516 (99.9)	411 (0.02)	1 997 200 (99.9)	1727 (0.1)
25.0-29.9	764 768 (99.9)	125 (0.02)	764 292 (99.9)	601 (0.1)
≥30.0	328 343 (99.9)	49 (0.01)	328 151 (99.9)	241 (0.1)
Unknown	845 849 (99.9)	277 (0.03)	844 949 (99.9)	1177 (0.1)
Diabetes before or during the index pregnancy				
No	6 648 443 (99.9)	8616 (0.1)	6 644 062 (99.8)	12 997 (0.2)
Yes	101 453 (99.9)	48 (0.1)	101 404 (99.9)	97 (0.1)
Hypertensive disease before or during the index pregnancy				
No	6 514 941 (99.9)	8477 (0.1)	6 510 707 (99.8)	12 711 (0.2)
Yes	234 955 (99.9)	187 (0.1)	234 759 (99.8)	383 (0.2)
Psychiatric disorders before or during the index pregnancy				
No	6 454 410 (99.9)	8540 (0.1)	6 450 149 (99.8)	12 801 (0.2)
Yes	295 486 (99.9)	124 (0.1)	295 317 (99.9)	293 (0.1)
Family history of cardiovascular disease before the index birth				
No	3 642 037 (99.9)	5836 (0.1)	3 640 457 (99.8)	7416 (0.2)
Yes	3 107 859 (99.9)	2828 (0.1)	3 105 009 (99.8)	5678 (0.2)

Abbreviation: BMI, body mass index (calculated as weight in kilograms divided by height in meters squared).

^a^ Birth weight below the 10th percentile of the gestational age and sex-specific standard fetal growth curve.

^b^ Information on maternal smoking has been available since 1991 in Denmark and since 1982 in Sweden.

^c^ Information on maternal BMI has been available since 2003 in Denmark and since 1982 in Sweden.

### Maternal Bereavement and Offspring IHD Risk

**Figure 1.**
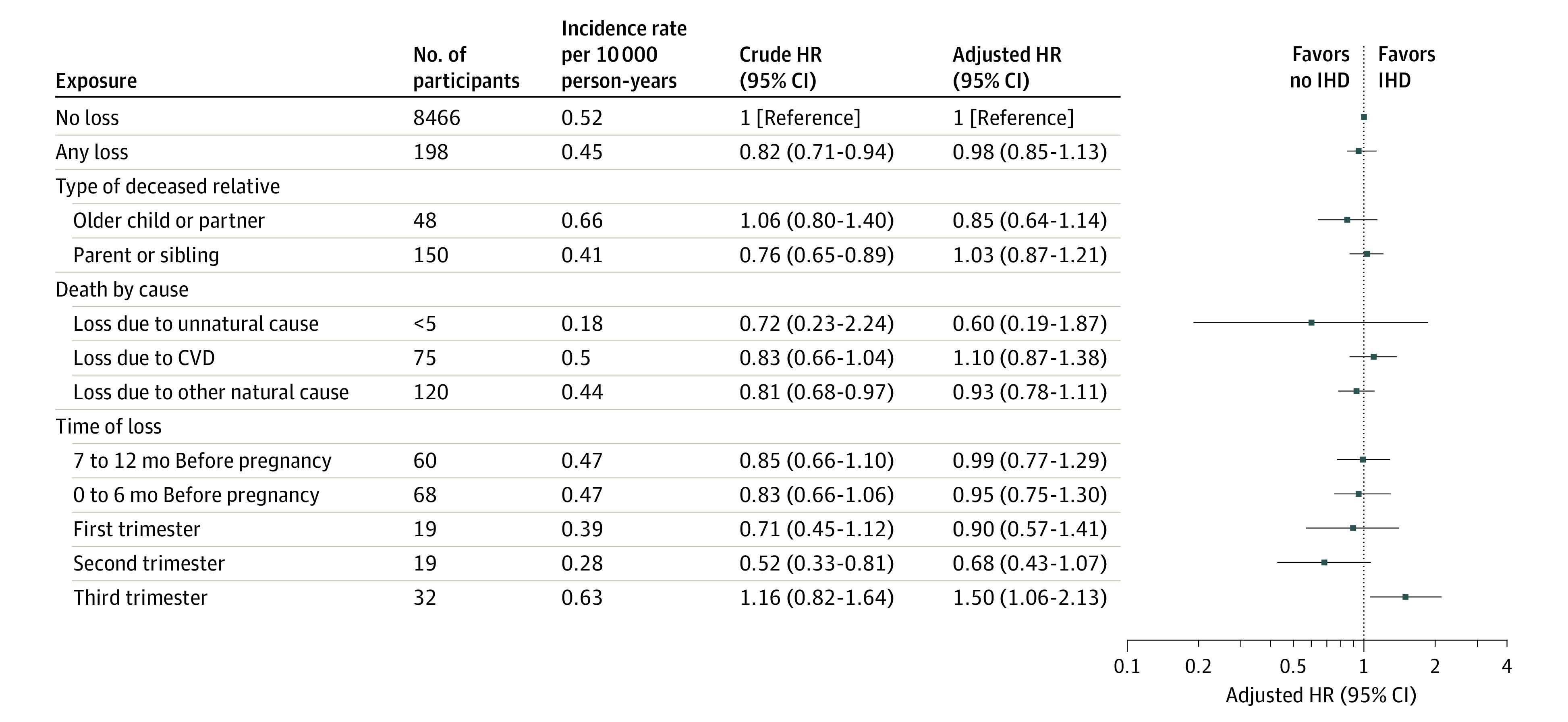
Hazard Ratios (HRs) for Ischemic Heart Disease (IHD) in the Offspring According to Maternal Bereavement Findings were adjusted for sex, country, and year of birth; maternal country of origin, parity, age, educational level, and marital status at the time of birth; maternal hypertensive disease, diabetes, and psychiatric disorders before the index birth; and family history of cardiovascular disease (CVD).

The IHD incidence rate for exposed offspring was 0.52 per 10 000 person-years and, for unexposed offspring, 0.45 per 10 000 person-years (Figure 1; eFigure 1 in Supplement 1). Maternal loss of any relative the year before or during pregnancy was not associated with the risk of IHD in the offspring (adjusted HR [AHR], 0.98; 95% CI, 0.85-1.13) (Figure 1). Furthermore, there was no association between bereavement when categorized by the type of decreased relative, with AHRs 0.85 (95% CI, 0.64-1.14) in the case of loss of older child or partner and 1.03 (95% CI, 0.87-1.21) in the case of loss of parent or sibling or the relative's cause of death, with AHRs 0.60 (95% CI, 0.19-1.87) in the case of loss due to unnatural cause, 1.10 (95% CI, 0.87-1.38) in the case of loss due to CVD, and 0.93 (95% CI, 0.78-1.11) in the case of loss due to other natural cause and the risk of IHD. However, when exposure was categorized according to the time of bereavement, exposure in the third trimester was associated with an increased risk of IHD (AHR, 1.50; 95% CI, 1.06-2.13).

### Maternal Bereavement and Offspring Stroke Risk

**Figure 2.**
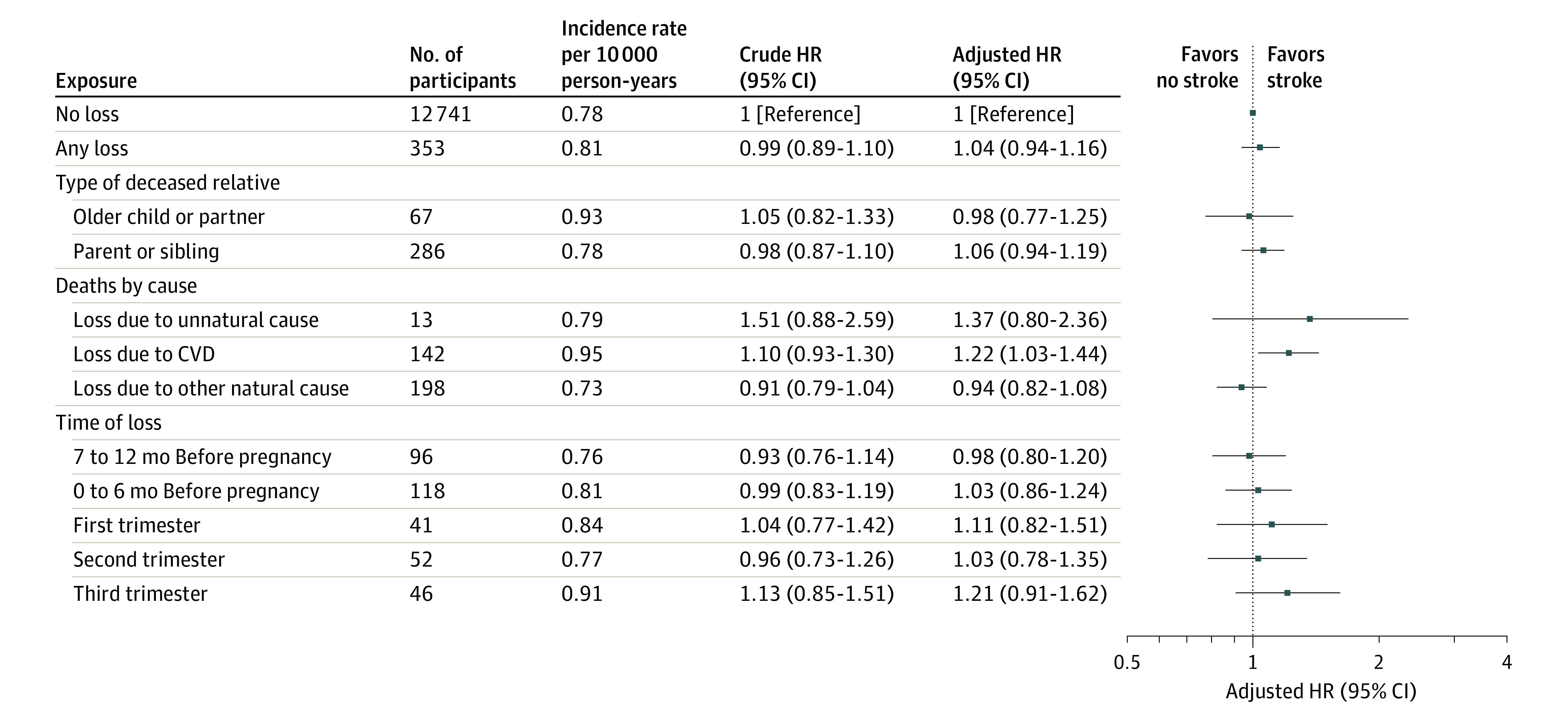
Hazard Ratios (HRs) for Stroke in the Offspring According to Maternal Bereavement Findings were adjusted for sex, country, and year of birth; maternal country of origin, parity, age, educational level, and marital status at the time of birth; maternal hypertensive disease, diabetes, and psychiatric disorders before the index birth; and family history of cardiovascular disease (CVD).

The incidence rate for stroke among the exposed offspring was 0.81 per 10 000 person-years and, for unexposed offspring, 0.78 per 10 000 person-years (Figure 2; eFigure 2 in Supplement 1). Maternal bereavement was not associated with stroke or either of its 2 subtypes; the AHRs were 1.04 (95% CI, 0.94-1.16) for stroke, 1.11 (95% CI, 0.97-1.27) for ischemic stroke, and 0.95 (95% CI, 0.77-1.18) for hemorrhagic stroke (Figure 2; eTable 4 in Supplement 1). When we categorized exposure by the relative’s cause of death, we found that offspring whose mother lost a relative due to CVD had higher risks of stroke (AHR, 1.22; 95% CI, 1.03-1.44) and ischemic stroke (AHR, 1.41; 95% CI, 1.14-1.73) than the unexposed cohort. We found no association between bereavement by the type of decreased relative, with AHRs 0.98 (95% CI, 0.77-1.25) in the case of loss of older child or partner and 1.06 (95% CI, 0.94-1.19) in the case of loss of parent or sibling or by the relative's time of death, with AHRs 0.98 (95% CI, 0.80-1.20) in the case of loss during 7 to 12 months before pregnancy, 1.03 (95% CI, 0.86-1.24) in the case of loss 6 months before pregnancy, 1.11 (95% CI, 0.82-1.51) in the case of loss during the first trimester, 1.03 (95% CI, 0.78-1.35) in the case of loss during the second trimester, and 1.21 (95% CI, 0.91-1.62) in the case of loss during the third trimester.

The differences between maternal bereavement the year before or during pregnancy and the offspring IHD and stroke risks did not differ substantially according to the offspring attained age, sex, country, or year of birth (eTable 3 and eTables 5-7 in Supplement 1).

### Sensitivity Analyses

Because we observed an association between maternal bereavement during the third trimester of pregnancy and an increased risk of IHD, we repeated the corresponding analyses. The association between maternal bereavement in the third trimester and the risk of IHD did not substantially change after we excluded offspring with preterm birth, small for gestational age, or CHD (Table 2). When we included maternal smoking or BMI during early pregnancy in the main model among individuals with complete information on these 2 covariates, the results did not change substantially (eTable 8 in Supplement 1).

**Table 2.** Ischemic Heart Disease According to Maternal Bereavement During the Third Trimester

Variable	No. of events	Incidence rate, per 10 000 person-years	HR (95% CI)	
			Model 1^a^	Model 2^b^
**Without preterm birth**				
Unexposed	5132	0.37	1.0 [Reference]	1.0 [Reference]
Any loss during the third trimester	29	0.61	1.50 (1.04-2.16)	1.62 (1.12-2.33)
**Without small for gestational age**				
Unexposed	4445	0.34	1.0 [Reference]	1.0 [Reference]
Any loss during the third trimester	23	0.53	1.41 (0.94-2.13)	1.52 (1.01-2.29)
**Without congenital anomalies**				
Unexposed	7097	0.48	1.0 [Reference]	1.0 [Reference]
Any loss during the third trimester	27	0.59	1.16 (0.79-1.69)	1.52 (1.04-2.21)

Abbreviation: HR, hazard ratio.

^a^ Model 1 was unadjusted.

^b^ Model 2 was adjusted for sex, country, and year of birth; maternal country of origin, parity, age, educational level, and marital status at the time of birth; maternal hypertensive disease, diabetes, and psychiatric disorders before the index birth; and family history of cardiovascular disease.

## Discussion

In this large cohort study including 6.8 million live births that were followed up to 5 decades, overall, we did not observe associations or clinically meaningful findings between maternal loss of any close relative the year before or during pregnancy and the risks of IHD and stroke in the offspring. Similarly, we found no associations when we studied the most severe forms of exposure, ie, loss of a child or partner and loss by unnatural death. However, we found an association between maternal bereavement in the third trimester and an increased risk of IHD in the offspring.

### Comparison With Previous Studies

Our findings are consistent with some but not all previous studies investigating the role of prenatal stress in the etiology of IHD and stroke. Of the studies that used data from the Dutch Famine Birth Cohort (1944-1945) with a follow-up of up to 58 years, 2 reported an association between prenatal exposure to famine and an increased risk of IHD,^17,18^ while 1 study found no association.^19^ In contrast, the Helsinki Birth Cohort Study observed an inverse association between prenatal exposure to bombings and the risk of both IHD and cerebrovascular disease in women, whereas corresponding findings in men were ambiguous.^20^ Inconsistencies in earlier findings may be attributed to the limitation of previous studies in measuring exposure at an ecologic level rather than at the individual level, differences in the duration of follow-up, lack of information on important confounders, and survival bias, given that those who survived bombings or hunger may be a healthier group. In addition, it is difficult to separate the direct effect of stress from that of malnutrition, poor housing conditions, and limited access to health care during famine or war. A study using a cohort partly overlapping with our Danish cohort and following 2.6 million study participants up to the age of 40 years found a 13% increased risk of overall CVD associated with maternal bereavement the year before or during pregnancy.^21^ However, this association disappeared in sibling analysis, suggesting that familial confounding mostly explained the observed association. Nevertheless, this Danish study had limited statistical power to thoroughly examine whether associations between bereavement and specific CVDs, such as IHD and stroke, were present. Our large sample size and the nearly 5 decades of follow-up allowed us to study maternal stress and the risks of IHD and stroke with better precision and in more detail than in the previous studies. First, as information on exposure was collected from high-quality mortality registers, before and independently of the outcomes, we avoided recall and selection bias. Moreover, the categorization of exposure by severity of bereavement allowed us to explore dose-response relationships, while categorization of exposure by timing of loss allowed us to explore the role of stress in specific periods of fetal development. Second, unlike the previous famine and wartime studies with older individuals, our study evaluated risks of IHD and stroke from childhood to mid-adulthood. Third, the extensive linkages to 2 nationwide registers allowed us to control for a larger number of potential confounders than in several previous studies.^17,18,19,20,21^

### Interpretations of the Main Findings

In contrast to findings from previous studies,^17,18,21^ we did not find evidence for an association between maternal bereavement and the risks of IHD and stroke in the study cohort. We speculate that one reason for the lack of associations relates to the short follow-up period. Previous studies have reported increased risks of some CVD risk factors, such as obesity,^28^ hypertension,^21^ and diabetes,^29^ following prenatal stress exposure; it is possible that exposure to these intermediate factors may need to be longer to lead to clinically manifested IHD or stroke. In addition, our finding that maternal bereavement was associated with stroke only when the relative died of CVD, but not when the loss was due to other causes, is supportive of the hypothesis that the association is explained rather by confounding due to cardiovascular risk factors that cluster in the family than by stress-related mechanisms.

We did not have a clearly predefined hypothesis about the timing of stress and IHD and stroke risk, and through exploratory analysis, we found an association with stress only in the third trimester of pregnancy and IHD. One potential explanation is that stress during the third trimester may affect fetal growth and gestational age or induce cardiovascular remodeling, which could predispose the offspring to IHD later in life. Earlier studies suggesting that children born to mothers with bereavement have increased risks of preterm birth,^10^ low birth weight,^9^ and CHD^30^ support this explanation. However, the association persisted even after excluding preterm birth, fetal growth restriction, or CHD, indicating that stress during the third trimester may be involved in the development of IHD through other pathways. The number of IHD cases among those exposed in the third trimester was small. Therefore, further research with larger samples is needed to confirm our findings and understand the underlying mechanisms.

### Limitations

The study has several limitations. First, although the date of maternal bereavement after a relative’s death is highly reliable, it may not reflect the precise onset of stress exposure. The stress of the exposed individual may have started earlier, eg, at the time of diagnosis of a fatal disease. Caution is thus needed when interpreting the trimester-specific association for stress. Second, although our study population included all live births in 2 countries across 5 decades, due to the relatively low prevalence of our exposure and outcomes, statistical power may have been limited to detect associations, especially in analyses involving subcategories of the exposure. Third, our findings may only be generalized to individuals living in countries with free universal health care systems and a developed welfare system similar to those of Denmark and Sweden.

## Conclusion

The findings of this cohort study suggest that prenatal stress may not be associated with the risk of IHD and stroke in offspring in the first 5 decades of life. Our finding of an association between stress during the third trimester of pregnancy and an increased risk of IHD warrants further investigation.

## Notes

Supplement 1. **eTable 1.** Description of Danish and Swedish Registers Used in This Study **eTable 2.** *International Classification of Diseases* Codes for Exposure, Outcome, and Covariates in Our Study **eTable 3.** Incidence Rates and Hazard Ratios With 95% Confidence Intervals for Ischemic Heart Disease and Stroke According to Maternal Bereavement, Stratified by the Offspring’s Attained Age **eTable 4.** Hazard Ratios and 95% Confidence Intervals for Stroke Subtypes in the Offspring According to Maternal Bereavement **eTable 5.** Hazard Ratios and 95% Confidence Intervals for Ischemic Heart Disease and Stroke According to Maternal Bereavement, Stratified by the Offspring’s Sex **eTable 6.** Hazard Ratios and 95% Confidence Intervals for Ischemic Heart Disease and Stroke According to Maternal Bereavement, Stratified by Study Country **eTable 7.** Hazard Ratios and 95% Confidence Intervals for Ischemic Heart Disease and Stroke According to Maternal Bereavement, Stratified by Calendar Year of Birth **eTable 8.** Hazard Ratios and 95% Confidence Intervals for Ischemic Heart Disease and Stroke According to Maternal Bereavement, in the Offspring With Information on Maternal Smoking and Body-Mass Index During Early Pregnancy **eFigure 1.** Cumulative Incidence of Ischemic Heart Disease According to Maternal Bereavement **eFigure 2.** Cumulative Incidence of Stroke According to Maternal Bereavement

Supplement 2.
Data Sharing Statement
