# Error in Author’s Name

**DOI:** 10.1001/jamanetworkopen.2024.0490

**Published:** 2024-02-02

In the Original Investigation titled “Prenatal Exposure to Severe Stress and Risks of Ischemic Heart Disease and Stroke in Offspring,”^1^ published December 27, 2023, there was a spelling error in an author name. The spelling of the fourth author “Jing Li, MD, PhD,” should have been “Jiong Li, MD, PhD.” This article was corrected.^1^
