# Supplementary material for: Prenatal Exposure to Severe Stress and Risks of Ischemic Heart Disease and Stroke in Offspring
Source: JAMA Netw Open. 2023 Dec 27;6(12):e2349463. doi: 10.1001/jamanetworkopen.2023.49463 (PMC10753395; doi:10.1001/jamanetworkopen.2023.49463)
Supplement: Supplement 1. — eTable 1. Description of Danish and Swedish Registers Used in This Study eTable 2. International Classification of Diseases Codes for Exposure, Outcome, and Covariates in Our Study eTable 3. Incidence Rates and Hazard Ratios With 95% Confidence Intervals for Ischemic Heart Disease and Stroke According to Maternal Bereavement, Stratified by the Offspring’s Attained Age eTable 4. Hazard Ratios and 95% Confidence Intervals for Stroke Subtypes in the Offspring According to Maternal Bereavement eTable 5. Hazard Ratios and 95% Confidence Intervals for Ischemic Heart Disease and Stroke According to Maternal Bereavement, Stratified by the Offspring’s Sex eTable 6. Hazard Ratios and 95% Confidence Intervals for Ischemic Heart Disease and Stroke According to Maternal Bereavement, Stratified by Study Country eTable 7. Hazard Ratios and 95% Confidence Intervals for Ischemic Heart Disease and Stroke According to Maternal Bereavement, Stratified by Calendar Year of Birth eTable 8. Hazard Ratios and 95% Confidence Intervals for Ischemic Heart Disease and Stroke According to Maternal Bereavement, in the Offspring With Information on Maternal Smoking and Body-Mass Index During Early Pregnancy eFigure 1. Cumulative Incidence of Ischemic Heart Disease According to Maternal Bereavement eFigure 2. Cumulative Incidence of Stroke According to Maternal Bereavement [file jamanetwopen-e2349463-s001.pdf]

Supplementary Online Content

Yang F, Janszky I, Roos N, Li J, László KD. Prenatal exposure to severe stress and risks of ischemic heart disease and stroke in offspring. *JAMA Netw Open*. 20236(12):e2349463. doi:10.1001/jamanetworkopen.2023.49463

eTable 1. Description of Danish and Swedish Registers Used in This Study

eTable 2. *International Classification of Diseases* Codes for Exposure, Outcome, and Covariates in Our Study

eTable 3. Incidence Rates and Hazard Ratios With 95% Confidence Intervals for Ischemic Heart Disease and Stroke According to Maternal Bereavement, Stratified by the Offspring's Attained Age

eTable 4. Hazard Ratios and 95% Confidence Intervals for Stroke Subtypes in the Offspring According to Maternal Bereavement

eTable 5. Hazard Ratios and 95% Confidence Intervals for Ischemic Heart Disease and Stroke According to Maternal Bereavement, Stratified by the Offspring's Sex

eTable 6. Hazard Ratios and 95% Confidence Intervals for Ischemic Heart Disease and Stroke According to Maternal Bereavement, Stratified by Study Country

eTable 7. Hazard Ratios and 95% Confidence Intervals for Ischemic Heart Disease and Stroke According to Maternal Bereavement, Stratified by Calendar Year of Birth

eTable 8. Hazard Ratios and 95% Confidence Intervals for Ischemic Heart Disease and Stroke According to Maternal Bereavement, in the Offspring With Information on Maternal Smoking and Body-Mass Index During Early Pregnancy

eFigure 1. Cumulative Incidence of Ischemic Heart Disease According to Maternal Bereavement

eFigure 2. Cumulative Incidence of Stroke According to Maternal Bereavement

This supplementary material has been provided by the authors to give readers additional information about their work.

© 2023 Yang F et al. *JAMA Network Open.*

eTable 1. Description of Danish and Swedish registers used in this study

Registers	Information
**Denmark**	
Danish Civil Registration System	The register was established in 1968. We extracted from this register data on individuals' sex, date and place of birth, vital status, marital status, migration, and information on parents and siblings.
Danish Integrated Database for Labour Market Research	The register was established in 1980 and includes information on age and completed educational attainment.
Danish Medical Birth Register	The register was established in 1973. We extracted from this register data on the index person's date of birth, sex, gestational age, birth weight, singleton/multiple birth, and their mothers' body-mass index (available since 2003) and smoking in early pregnancy (available since 1991), age at delivery and parity.
Danish National Patient Register	The register includes data on inpatient care (established in 1977) and outpatient care and emergency department contacts (established in 1995). We retrieved from this register information on the diseases of interest (International Classification of Disease codes and date).
Danish Register of Causes of Death	The register was established in 1970 and includes all Danish residents' date and cause of death.
**Sweden**	
Swedish Total Population Register	The register was established in 1968. We retrieved from this register information on individuals' sex, birth date, place of birth, civil status, marital status, and migration.

Swedish Multi-Generation Register	The register was established in 1961 and contains information on family relationships for all Swedish residents born after 1932.
Swedish Register of Education	The register was established in 1985 and contains yearly updated information on educational attainment for all Swedish residents aged 16-74 years.
Swedish Medical Birth Register	The register was established in 1973. We extracted from this register data on individuals' birth date, sex, gestational age, birth weight, singleton status, and their mothers' age at delivery, parity, medical diagnoses, and smoking and body-mass index in early pregnancy (available since 1982).
Swedish Patient Register	The register contains information on inpatient care (since1968, with nationwide coverage since 1987) and outpatient care (established in 2001). We retrieved from this register information on diagnoses and their dates.
Swedish Cause of Death Register	The register was established in 1952 and includes information on date and cause of death for all Swedish residents.

© 2023 Yang F et al. *JAMA Network Open.*

eTable 2. ***International Classification of Diseases*** **codes for exposure, outcome, and covariates in our study**

	ICD-8	ICD-9	ICD-10
Exposure			
**Unnatural death**			
Denmark	795, E800-E999		R95-R97, V00-Y99
Sweden	7959, 79621, E800-E999	798, E800-E999	R95, R96, R98, V01-Y98
**Death due to cardiovascular disease**			
Denmark	390-458		I00-I99
Sweden	390-458	390-459	I00-I99
**Other natural death**			
Denmark	All the other codes		All the other codes
Sweden	All the other codes	All the other codes	All the other codes
Outcome			
**Ischemic heart disease**			
Denmark	410-414		I20-I25
Sweden	410-414	410-414	I20-I25
**Stroke**			
Denmark	430, 431, 433, 434, 436		I60, I61, I63, I64
Sweden	430, 431, 433, 434, 436	430, 431, 433, 434, 436	I60, I61, I63, I64
Covariates			
**Overall cardiovascular disease**			
Denmark	390-458		I00-I99
Sweden	390-458	390-459	I00-I99
**Hypertension**			

© 2023 Yang F et al. *JAMA Network Open.*

Denmark	400-404		I10-I15, O10, O11
Sweden	400-404	401-405, 642A, 642B, 642C, 642H	I10-I15, O10, O11
**Congenital heart disease**			
Denmark	746, 747		Q20-Q27
Sweden	746, 747	745, 746, 747	Q20-Q28
**Diabetes**			
Denmark	249, 250		E10-E14, O24
Sweden	250	250	E10-E14, O24
**Psychiatric disorders**			
Denmark	290-315		F00-F99
Sweden	290-315	290-319	F00-F99

© 2023 Yang F et al. *JAMA Network Open.*

eTable 3. Incidence rates and hazard ratios with 95% confidence intervals for ischemic heart disease and stroke according to maternal bereavement, stratified by the offspring's attained age

Exposure	Number of events	Incidence rate, per 10,000 person-years	HR (95% CI)	
			Model 1 [a]	Model 2 [b]
**Ischemic heart disease**				
**Attained age <18 years**				
No loss	622	0.06	1.0 (Reference)	1.0 (Reference)
Any loss	19	0.07	1.16 (0.73-1.83)	1.14 (0.72-1.80)
**18≤Attained age ≤30 years**				
No loss	2275	0.56	1.0 (Reference)	1.0 (Reference)
Any loss	56	0.49	0.88 (0.67-1.15)	1.01 (0.78-1.32)
**Attained age >30 years**				
No loss	5569	2.69	1.0 (Reference)	1.0 (Reference)
Any loss	123	2.07	0.76 (0.63-0.90)	0.95 (0.79-1.13)
**Stroke**				
**Attained age <18 years**				
No loss	1330	0.13	1.0 (Reference)	1.0 (Reference)
Any loss	30	0.11	0.85 (0.59-1.22)	0.85 (0.59-1.22)
**18≤Attained age ≤30 years**				
No loss	2539	0.58	1.0 (Reference)	1.0 (Reference)
Any loss	65	0.57	0.98 (0.77-1.26)	0.97 (0.76-1.24)
**Attained age >30 years**				
No loss	9052	4.38	1.0 (Reference)	1.0 (Reference)
Any loss	258	4.35	0.94 (0.83-1.06)	1.10 (0.97-1.24)

Abbreviations: HR, hazard ratio; CI, confidence interval.

© 2023 Yang F et al. *JAMA Network Open.*

[a] Model 1 was unadjusted.

[b] Model 2 was adjusted for sex, country and calendar year of birth, maternal country of origin, parity, age, education and marital status at the time of birth, hypertensive disease, diabetes, and psychiatric disorders before the index birth, and family history of cardiovascular diseases.

© 2023 Yang F et al. *JAMA Network Open.*

eTable 4. Hazard ratios and 95% confidence intervals for stroke subtypes in the offspring according to maternal bereavement

Exposure	Number of events	Incidence rate, per 10 000 person-years	HR (95% CI)	
			Model 1 [a]	Model 2 [b]
**Ischemic stroke**				
No loss	7145	0.44	1.0 (Reference)	1.0 (Reference)
Any loss	207	0.47	1.03 (0.90-1.18)	1.11 (0.97-1.27)
**Type of deceased relative**				
Older child or partner [c]	43	0.59	1.17 (0.87-1.58)	1.10 (0.81-1.48)
Parent or sibling [d]	164	0.45	0.99 (0.85-1.16)	1.11 (0.95-1.30)
**Deaths by cause**				
Loss due to unnatural cause	8	0.49	1.79 (0.90-3.58)	1.54 (0.77-3.09)
Loss due to CVD	90	0.60	1.22 (0.99-1.50)	1.41 (1.14-1.73)
Loss due to other natural cause	109	0.40	0.89 (0.73-1.07)	0.93 (0.77-1.12)
**Time of loss**				
7 to 12 months before pregnancy	56	0.44	0.96 (0.74-1.25)	1.03 (0.79-1.34)
0 to 6 months before pregnancy	67	0.46	0.99 (0.78-1.27)	1.05 (0.83-1.34)
First trimester	26	0.53	1.17 (0.80-1.72)	1.28 (0.87-1.89)
Second trimester	35	0.52	1.15 (0.82-1.60)	1.26 (0.91-1.76)
Third trimester	23	0.45	1.00 (0.67-1.51)	1.11 (0.73-1.67)

© 2023 Yang F et al. *JAMA Network Open.*

**Hemorrhagic stroke**				
No loss	3202	0.20	1.0 (Reference)	1.0 (Reference)
Any loss	83	0.19	0.94 (0.75-1.17)	0.95 (0.77-1.18)
**Type of deceased relative**				
Older child or partner [c]	15	0.21	0.96 (0.58-1.60)	0.95 (0.57-1.58)
Parent or sibling [d]	68	0.19	0.93 (0.73-1.18)	0.95 (0.75-1.21)
**Deaths by cause**				
Loss due to unnatural cause	<5	0.06	-	-
Loss due to CVD	33	0.22	1.04 (0.74-1.47)	1.08 (0.77-1.53)
Loss due to other natural cause	49	0.18	0.90 (0.68-1.19)	0.91 (0.68-1.20)
**Time of loss**				
7 to 12 months before pregnancy	23	0.18	0.93 (0.76-1.14)	0.98 (0.80-1.20)
0 to 6 months before pregnancy	30	0.21	0.99 (0.83-1.19)	1.03 (0.86-1.24)
First trimester	12	0.24	1.04 (0.77-1.42)	1.11 (0.82-1.51)
Second trimester	6	0.09	0.96 (0.73-1.26)	1.03 (0.78-1.35)
Third trimester	12	0.24	1.13 (0.85-1.51)	1.21 (0.91-1.62)

Abbreviations: HR, hazard ratio; CI, confidence interval.

[a] Model 1 was unadjusted.

[b] Model 2 was adjusted for sex, country and calendar year of birth, maternal country of origin, parity, age, education and marital status at the time of birth, hypertensive disease, diabetes, and psychiatric disorders before the index birth, and family history of cardiovascular diseases.

[c] Analyses restricted to children whose mother had register links to her partner or at least a live older child at the start of the exposure period (N= 6 755 410).

© 2023 Yang F et al. *JAMA Network Open.*

[d] Analyses restricted to children whose mother had at least a live parent or a live sibling in the registers at the start of the exposure period (N= 6 749 403).

© 2023 Yang F et al. *JAMA Network Open.*

eTable 5. Hazard ratios and 95% confidence intervals for ischemic heart disease and stroke according to maternal bereavement, stratified by the offspring's sex

	Number of events	Incidence rate, per 10 000 person-years	HR (95% CI)	
			Model 1 [a]	Model 2 [b]
**Boy (N=3 472 441)**				
**Ischemic heart disease**				
No loss	5612	0.67	1.0 (Reference)	1.0 (Reference)
Any loss	122	0.54	0.76 (0.64-0.91)	0.91 (0.76-1.09)
**Stroke**				
No loss	6716	0.80	1.0 (Reference)	1.0 (Reference)
Any loss	178	0.79	0.95 (0.82-1.10)	0.99 (0.85-1.15)
**Girl (N=3 286 119)**				
**Ischemic heart disease**				
No loss	2854	0.36	1.0 (Reference)	1.0 (Reference)
Any loss	76	0.35	0.93 (0.74-1.17)	1.12 (0.89-1.40)
**Stroke**				
No loss	6025	0.76	1.0 (Reference)	1.0 (Reference)
Any loss	175	0.82	1.04 (0.89-1.20)	1.10 (0.95-1.28)

Abbreviations: HR, hazard ratio; CI, confidence interval.

[a] Model 1 was unadjusted.

[b] Model 2 was adjusted for country and calendar year of birth, maternal country of origin, parity, age, education and marital status at the time of birth, hypertensive disease, diabetes, and psychiatric disorders before the index birth, and family history of cardiovascular diseases.

© 2023 Yang F et al. *JAMA Network Open.*

The P-values for the interaction between maternal bereavement and sex on the risk of ischemic heart disease and stroke are 0.19 and 0.43, respectively.

© 2023 Yang F et al. *JAMA Network Open.*

eTable 6. Hazard ratios and 95% confidence intervals for ischemic heart disease and stroke according to maternal bereavement, stratified by study country

	Number of events	Incidence rate, per 10 000 person-years	HR (95% CI)	
			Model 1 [a]	Model 2 [b]
**Denmark (N=2 664 576)**				
**Ischemic heart disease**				
No loss	5016	0.90	1.0 (Reference)	1.0 (Reference)
Any loss	89	0.72	1.11 (0.90-1.37)	1.03 (0.83-1.27)
**Stroke**				
No loss	5441	0.97	1.0 (Reference)	1.0 (Reference)
Any loss	112	0.90	1.12 (0.93-1.35)	1.07 (0.89-1.29)
**Sweden (N=4 093 984)**				
**Ischemic heart disease**				
No loss	3450	0.32	1.0 (Reference)	1.0 (Reference)
Any loss	109	0.35	0.95 (0.78-1.14)	0.94 (0.77-1.13)
**Stroke**				
No loss	7300	0.68	1.0 (Reference)	1.0 (Reference)
Any loss	241	0.77	1.03 (0.91-1.17)	1.04 (0.91-1.18)

Abbreviations: HR, hazard ratio; CI, confidence interval.

[a] Model 1 was unadjusted.

[b] Model 2 was adjusted for sex and calendar year of birth, maternal country of origin, parity, age, education and marital status at the time of birth, hypertensive disease, diabetes, and psychiatric disorders before the index birth, and family history of cardiovascular diseases.

© 2023 Yang F et al. *JAMA Network Open.*

The P-values for the interaction between maternal bereavement and study country on the risk of ischemic heart disease and stroke are 0.25 and 0.57, respectively.

© 2023 Yang F et al. *JAMA Network Open.*

eTable 7. Hazard ratios and 95% confidence intervals for ischemic heart disease and stroke according to maternal bereavement, stratified by calendar year of birth

Calendar year of birth	Number of events	Incidence rate, per 10 000 person-years	HR (95% CI)	
			Model 1 [a]	Model 2 [b]
**1973-1994 (N=3 523 107)**				
**Ischemic heart disease**				
No loss	8135	0.69	1.0 (Reference)	1.0 (Reference)
Any loss	187	0.56	0.79 (0.69-0.92)	0.96 (0.83-1.12)
**Stroke**				
No loss	11037	0.94	1.0 (Reference)	1.0 (Reference)
Any loss	317	0.95	1.01 (0.90-1.12)	1.07 (0.96-1.20)
**1995-2016 (N=3 235 453)**				
**Ischemic heart disease**				
No loss	331	0.07	1.0 (Reference)	1.0 (Reference)
Any loss	11	0.11	1.43 (0.78-2.60)	1.36 (0.75-2.49)
**Stroke**				
No loss	1704	0.38	1.0 (Reference)	1.0 (Reference)
Any loss	36	0.35	0.91 (0.65-1.27)	0.90 (0.65-1.25)

Abbreviations: HR, hazard ratio; CI, confidence interval.

[a] Model 1 was unadjusted.

[b] Model 2 was adjusted for country, sex and calendar year of birth, maternal country of origin, parity, age, education and marital status at the time of birth, hypertensive disease, diabetes, and psychiatric disorders before the index birth, and family history of cardiovascular diseases.

© 2023 Yang F et al. *JAMA Network Open.*

The P-values for the interaction between maternal bereavement and calendar year of birth on the risk of ischemic heart disease and stroke are 0.86 and 0.33, respectively.

© 2023 Yang F et al. *JAMA Network Open.*

eTable 8. Hazard ratios and 95% confidence intervals for ischemic heart disease and stroke according to maternal bereavement, in the offspring with information on maternal smoking and body-mass index during early pregnancy

	Number of events	Incidence rate, per 10 000 person-years	HR (95% CI)		
			Model 1 [a]	Model 2 [b]	Model 3
**Restricted to offspring with complete data on maternal smoking (N=4 553 329) [c]**					
**Ischemic heart disease**					
No loss	1057	0.12	1.0 (Reference)	1.0 (Reference)	1.0 (Reference)
Any loss	35	0.15	1.14 (0.82-1.60)	1.13 (0.81-1.58)	1.12 (0.80-1.57)
**Stroke**					
No loss	4040	0.47	1.0 (Reference)	1.0 (Reference)	1.0 (Reference)
Any loss	120	0.52	1.05 (0.88-1.26)	1.05 (0.88-1.26)	1.05 (0.87-1.25)
**Restricted to offspring with complete data on maternal body-mass index (N=3 219 168) [d]**					
**Ischemic heart disease**					
No loss	595	0.11	1.0 (Reference)	1.0 (Reference)	1.0 (Reference)
Any loss	18	0.12	1.07 (0.67-1.70)	1.05 (0.66-1.68)	1.05 (0.66-1.68)
**Stroke**					
No loss	2646	0.47	1.0 (Reference)	1.0 (Reference)	1.0 (Reference)
Any loss	73	0.50	1.02 (0.80-1.28)	1.02 (0.81-1.29)	1.02 (0.80-1.28)

Abbreviations: HR, hazard ratio; CI, confidence interval.

[a] Model 1 was unadjusted.

[b] Model 2 was adjusted for sex, country and calendar year of birth, maternal country of origin, parity, age, education and marital status at the time of birth, hypertensive disease, diabetes, and psychiatric disorders before the index birth, family history of cardiovascular diseases.

© 2023 Yang F et al. *JAMA Network Open.*

[c] Model 3 was further adjusted for maternal smoking during early pregnancy in addition to factors included in Model 2.

[d] Model 3 was further adjusted for maternal body-mass index during early pregnancy in addition to factors included in Model 2.

© 2023 Yang F et al. *JAMA Network Open.*

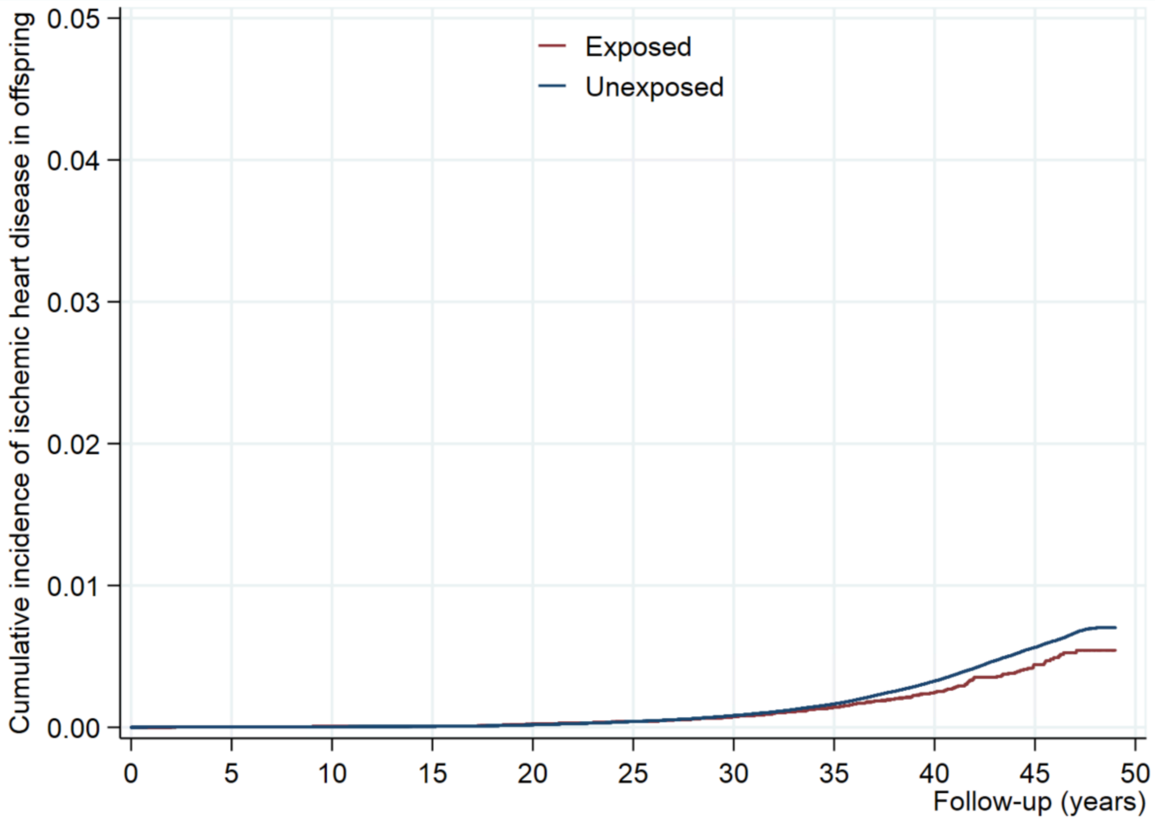

Number at risk

Exposed	167192	159699	146924	130098	111741	90956	67764	46007	28268	11790	0
Unexposed	6591368	6211051	5577748	4767571	3993419	3228094	2418041	1654309	979007	340802	0

eFigure 1. Cumulative incidence of ischemic heart disease according to maternal bereavement

The cumulative incidence plotted on the Y-axis ranges from 0 to 0.05.

© 2023 Yang F et al. *JAMA Network Open.*

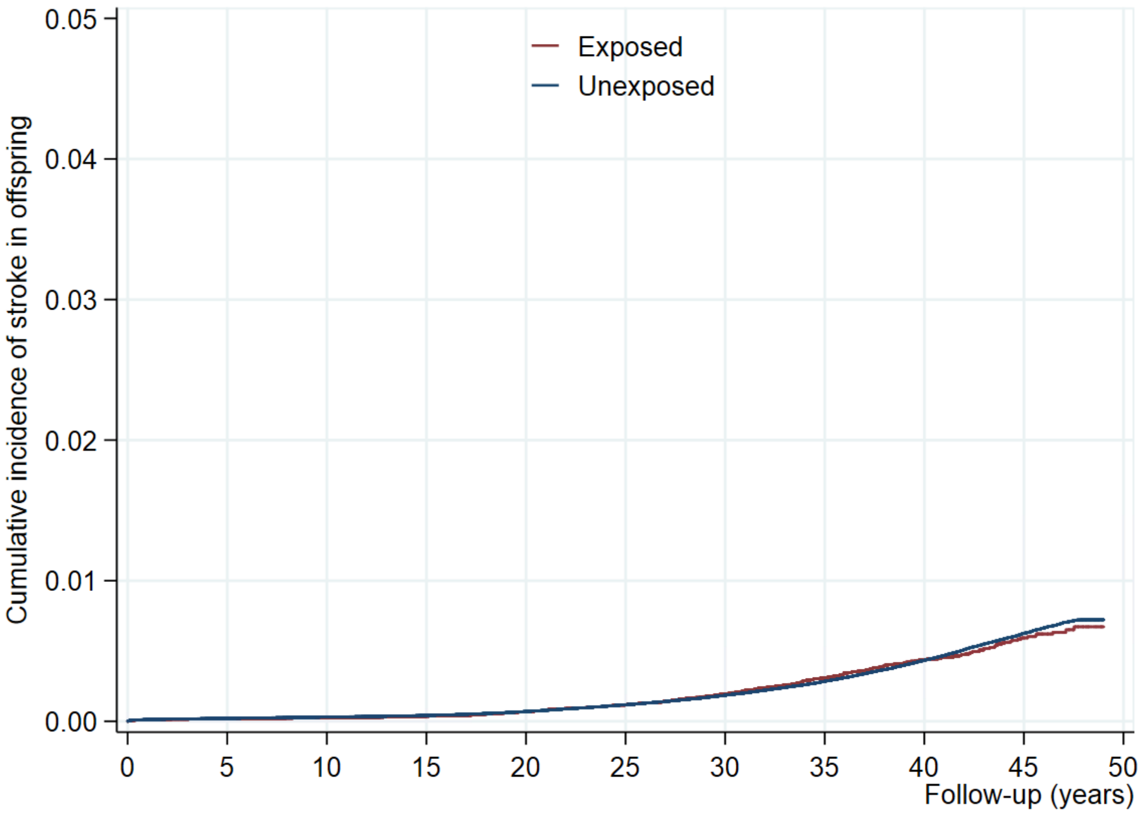

Number at risk

Exposed	167192	159674	146896	130066	111707	90890	67691	45931	28227	11767	0
Unexposed	6591368	6210135	5576673	4766341	3991821	3226088	2416042	1652775	978196	340385	0

eFigure 2. Cumulative incidence of stroke according to maternal bereavement

The cumulative incidence plotted on the Y-axis ranges from 0 to 0.05.

© 2023 Yang F et al. *JAMA Network Open.*
