# Supplementary material for: Prenatal Exposure to Severe Stress and Risks of Ischemic Heart Disease and Stroke in Offspring
Source: JAMA Netw Open. 2023 Dec 27;6(12):e2349463. doi: 10.1001/jamanetworkopen.2023.49463 (PMC10753395; doi:10.1001/jamanetworkopen.2023.49463)
Supplement: Supplement 2. — Data Sharing Statement [file jamanetwopen-e2349463-s002.pdf]

Data Sharing Statement

Yang. Prenatal Exposure to Severe Stress and Risks of Ischemic Heart Disease and Stroke in Offspring. *JAMA Netw Open*. Published December 27, 2023.
doi:10.1001/jamanetworkopen.2023.49463

Data
Data available: No

Additional Information
Explanation for why data not available: The datasets generated and/or analysed during the current study are not publicly available due to the data protection regulations. All datasets are stored on a secure server and may not be shared openly.
